# Occupational Styrene Exposure Induces Stress-Responsive Genes Involved in Cytoprotective and Cytotoxic Activities

**DOI:** 10.1371/journal.pone.0075401

**Published:** 2013-09-23

**Authors:** Elisabetta Strafella, Massimo Bracci, Sara Staffolani, Nicola Manzella, Daniele Giantomasi, Matteo Valentino, Monica Amati, Marco Tomasetti, Lory Santarelli

**Affiliations:** Department of Clinical and Molecular Sciences, Polytechnic University of Marche, Ancona, Italy; National Institutes of Health, United States of America

## Abstract

**Objective:**

The aim of this study was to evaluate the expression of a panel of genes involved in toxicology in response to styrene exposure at levels below the occupational standard setting.

**Methods:**

Workers in a fiber glass boat industry were evaluated for a panel of stress- and toxicity-related genes and associated with biochemical parameters related to hepatic injury. Urinary styrene metabolites (MA+PGA) of subjects and environmental sampling data collected for air at workplace were used to estimate styrene exposure.

**Results:**

Expression array analysis revealed massive upregulation of genes encoding stress-responsive proteins (HSPA1L, EGR1, IL-6, IL-1β, TNSF10 and TNFα) in the styrene-exposed group; the levels of cytokines released were further confirmed in serum. The exposed workers were then stratified by styrene exposure levels. EGR1 gene upregulation paralleled the expression and transcriptional protein levels of IL-6, TNSF10 and TNFα in styrene exposed workers, even at low level. The activation of the EGR1 pathway observed at low-styrene exposure was associated with a slight increase of hepatic markers found in highly exposed subjects, even though they were within normal range. The ALT and AST levels were not affected by alcohol consumption, and positively correlated with urinary styrene metabolites as evaluated by multiple regression analysis.

**Conclusion:**

The pro-inflammatory cytokines IL-6 and TNFα are the primary mediators of processes involved in the hepatic injury response and regeneration. Here, we show that styrene induced stress responsive genes involved in cytoprotection and cytotoxicity at low-exposure, that proceed to a mild subclinical hepatic toxicity at high-styrene exposure.

## Introduction

Styrene is a volatile organic compound used in factories for synthesis of plastic products. Human exposure occurs mainly in industrial settings such as hand-lamination plants, production of fiber glass-reinforced plastic products and in boats building [1]. Styrene and the primary metabolite styrene-7,8-oxide were found to be genotoxic and possibly carcinogenic [2,3,4], even at levels below the recommended TLV-TWAs (20 ppm) [5]. Nonetheless, epidemiologic studies have reported contradictory results. Workers exposed to styrene were found to have increased rates of mortality or incidences of lymphohematopoietic cancers, with suggestive evidence for pancreatic and esophageal tumors [6,7]. Long-term chemical carcinogenesis bioassays showed that styrene caused lung cancers in several strains of mice and mammary cancers in rats and styrene-7,8-oxide caused tumors of the forestomach in rats and mice and of the liver in mice. However, no coherent evidence that styrene exposure increases risk from cancers of the lymphatic and hematopoietic tissue, pancreas, or lung was found [8]. About 90% of inhaled styrene is absorbed by the lung and undergoes biotransformation to styrene-7,8-oxide via cytochrome P-450s, which is further metabolized to mandelic acid (MA) and phenylglyoxylic acid (PGA). Being metabolized by the liver, styrene-induced toxicity may result in hepatic injury. It was reported that styrene caused an increase in serum level of direct bilirubin and direct/total bilirubin ratio, indicating diminished hepatic clearance of conjugated bilirubin. A significant linear association between the alanine transaminase (ALT) and aspartate aminotransferase (AST) and exposure to styrene was found, with an increase in alkaline phosphatase (AP) in workers exposed above 25 ppm air styrene, suggesting the occurrence of hepatic damage probably due to styrene-induced oxidative stress [9]. Several forms of P-450, such as CYP2B1/2, CYP2E1, CYP3A2, and CYP1A2 have been suggested to generate hydroxyl radicals [10]. Although the toxic effect of styrene has been well documented, no adequate human studies are available for styrene-induced toxicity at levels below the occupational standard setting. In the present study, the expression of a panel of genes involved in the metabolism, oxidative stress, DNA damage and repair, carcinogenesis and cell death was evaluated in styrene-exposed fiber glass workers, assessing hepatic transaminase levels, which reflect active hepatic necrosis, and hepatic enzymes associated with cholestasis. 

## Materials and Methods

### Study population

Between February 2011 and May 2011, 96 workers who were occupationally exposed to styrene in the fiber glass boat industry were selected in the exposed group. The control group was composed of 54 office workers matched for age, gender and life style habits at the same workplace who had never been occupationally exposed to organic solvents. Workers with acute infections and/or diseases that may have suppressed their immune systems, those taking medication for a medical condition, and those with recent alcohol consumption more than 4 glasses/day were excluded from the study. The participants were interviewed by trained personnel and answered a detailed questionnaire on medical history and general characteristics including age, job characteristics including daily working hours and working duration, smoking, dietary, alcohol consumption and life style habits.

### Ethics statement

All subjects filled a questionnaire including their informed consent. The study was carried out according to the Helsinki Declaration and the samples were processed under approval of the written consent statement by Ethical Committee A.O.U. “Ospedali Riuniti” of Ancona, Italy (n° 211584), according to Ministerial Decree (DM 12/05/2006).

### Sample collection and analysis

The exposure to styrene was evaluated through environmental and biological monitoring.

Air sampling was conducted for 8h (8.00 a.m.to 4.00 p.m.) at three breathing zones after 3/4 consecutive working days. Air samples were collected on workers (n=15) belonging to two homogeneous groups.

Whole blood and urine samples were collected for both styrene-exposed and control subjects at the last day of the working week. The working day was 8h per day and 5 days per week. Whole blood was collected in fasting subject between 7:00 and 9.00 am, after centrifugation at 3000 rpm for 15 min serum was obtained and stored at -80°C for cytokine analysis. Blood samples collected into EDTA tubes were used for lymphocyte isolation. After blood centrifugation at 3000 g for 15 min, the buffy coat was removed, placed in a 15 ml Falcon tube and suspended in 4 ml of phosphate-buffered saline (PBS) buffer. The suspension was then layered onto 4 ml of Lympholyte-H (Cederlane, Hornby, Ontario, Canada) and centrifuged at 1000 g (20°C, 30 min). After centrifugation, the cloudy layer was collected and placed in a 15 ml Falcon tube, filled with PBS, pH 7.4, and centrifuged at 1000 g (20°C, 15 min). After removing the supernatant, the pellet was collected and stored at -80°C for RNA extraction. Urine samples were collected between 18:00-19:00 pm post-shift works and used for styrene metabolite analysis.

### Measuring ambient organic solvent levels

Ambient organic solvent levels were detected using Occupational Safety and Health Administration (OSHA 89) method [5]. Organic solvents in the air at the workplace were sampled in an activated charcoal tube (SKC 226-01, SKC, Eighty Four, PA, USA) connected to low-flow active samplers (Low Flow pump SKC, Eighty Four, PA, USA) and analyzed using gas chromatography (GC)-FID (TRACE GC 2000, ThermoQuest Instruments, MI, Italy).

### Analysis of Mandelic acid (MA) and Phenylglyoxylic acid (PGA)

Biological monitoring was carried out for all the participants. MA and PGA were analyzed in urine samples by HPLC-UV [11], and expressed as a function of creatinine (creat) levels (mg/g creat). MA and PGA are the main urinary metabolites that are used as biomarkers of exposure in biological monitoring of styrene exposure [5].

### Quantitative RT-PCR analysis

Total RNA was extracted from lymphocytes using PerfectPure RNA Kit (5Prime, Hamburg, Germany) according to the manufacturer’s instructions. The cDNA was synthesized using RT^2^-First Strand Kit (SA Biosciences, Frederick, MD, USA) according to the manufacturer’s instructions. Human Stress and Toxicity PathwayFinder™ RT^2^ Profiler™ PCR Expression Array (PAHS-003 SABiosciences) was used for gene expression profiling. The expression of 84 stress- and toxicity-related genes was assessed by qRT- PCR (Mastercycler EP Realplex, Eppendorf, Milano, Italy) using RT^2^ SYBR Green qPCR Master Mix (SABiosciences). Gene-specific product was normalized to GAPDH, and expressed as fold change (2^-Δ∆Ct^). The expression of selected genes was quantified using the TaqMan system (Applied Biosystems, Foster City, CA, USA), and the relative mRNA expression was calculated using the equation 2^-ΔCt^.

### Cytokine analysis

ELISA kits were used for serum quantification of human IL-6, IL-1β (Mabtech, Cincinnati, OH, USA), TNFSF10 (Biovendor, Brno, Czech Republic), and TNFα (Biosource international, Life Technologies, Camarillo, CA, USA) according to manufacturer’s instructions. Results were expressed as pg/ml.

### Hepatic biochemical parameters

Clinical hepatic parameters such as alanine transaminase (ALT), aspartate aminotransferase (AST), γ-glutamiltransferase (γ-GT), and alkaline phosphatase (AP), were measured in serum by standard clinical laboratory methods and expressed as U/L. The normal range for these hepatic biochemical parameters in the laboratory reference population were as follows: ALT > 40 U/L; AST > 37 U/L; γ-GT > 53 U/L; AP > 279 U/L.

### Statistical analysis

The normal distribution of all continuous variables was evaluated by Kolmogorov-Smirnov goodness-of-fit test. Since the variables did not show a normal distribution all results were presented as the median (25° percentile-75° percentile). Multiple comparisons and difference between groups were evaluated by the Kruskal-Wallis and Mann-Whitney tests, respectively. The Chi-square (χ^2^) test was used to test dichotomous or categorical parameters. Correlations were performed according to Spearman. Multiple linear regression analysis was used to model specific gene expression and hepatic parameters (dependent variables) as function of styrene exposure taking into account age, gender, body mass index, ethnicity, smoking, drinking and duration of exposure as possible covariates. The data were analyzed by the Statistical Package Social Sciences (version 19) software (SPSS, Chicago, IL, USA) and p-values less than 0.05 were considered significant.

## Results

Workers in a fiber glass boat industry were evaluated for a panel of stress- and toxicity-related genes and associated with biochemical parameters related to hepatic injury. The styrene-exposed workers showed significant ethnic diversity compared to control group. While, there were no significant differences in age, smoking, drinking rate, dietary habits, and anthropometric parameters between the two groups. Ambient exposure levels of styrene (median [25°-75° percentile], 50.7 [40.2-55.1] mg/m^3^) and the post-shift concentration of urinary styrene metabolites (MA+PGA, median [25°-75° percentile], 137.8 [80.3-266.4] mg/g creat) were both below the threshold limit value-time weighted average (TLV-TWA) fixed at 85 mg/m^3^ and 400 mg/g creat for ambient and individual styrene exposure, respectively [12].

**Figure 1:**
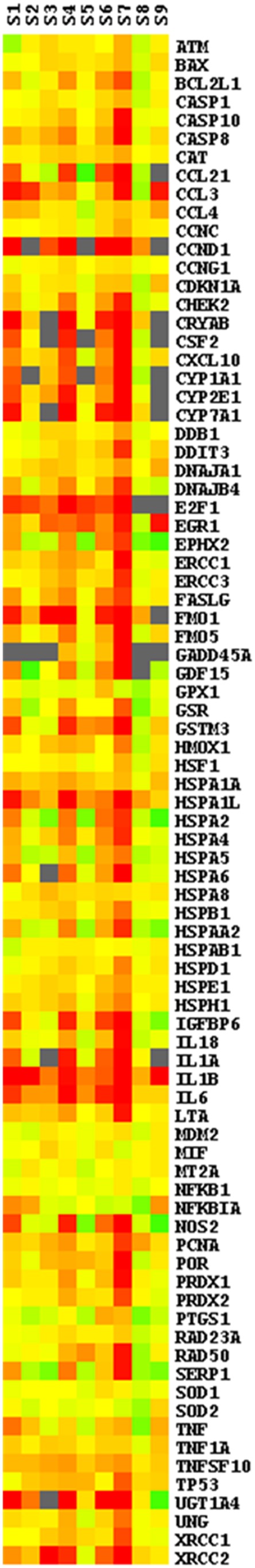
Hierarchical cluster analysis of gene expression. Gene expression in lymphocytes of 9 styrene exposed subjects is shown with respect to the pooled genes from lymphocytes of 5 non-exposed subjects. Genes were considered differentially expressed if their levels increased or decreased by more than 2-fold. Relative normalized expression for each gene is represented by color intensity (green, downregulation; yellow, no change in expression; red, increased expression; black, miRNA not detected).

**Table 1:** Demographic characteristics of styrene exposed and control groups.

	Control group (n=54)			Low-styrene group (n=51)			High-styrene group (n=45)		
	median	25°-75°	%	median	25°-75°	%	median	25°-75°	%
Age (years)	46.0	27.0-62.0		38.0	23.0-53.0		42.0	22.0-62.0	
Gender									
male			87.5			91.8			85.2
female			12.5			8.2			14.8
Body mass index (Kg/m^2^)	25.6	20.2-38.1		25.6	20.2-38.1		23.5	18.5-34.9	
Ethnicity (white)			100			78*			78*
Smoking									
no			65.5			63.3			66.7
yes			34.5			36.7			33.3
Drinking (glasses/day)	1.0	0.0-4.0		0.0	0.0-4.0		0.0	0.0-2.0	
0			30.3			82.4*			75.9*
1			67.9			13.7*			24.1*
4			1.8			3.9			0.0
Exposure duration (years)	-			6.0	2.0-36.0		5.0	3.0-11.0	
MA+PGA (mg/g creat)	0.4	0.0-2.0		94.5*	44.2-130		301.9*	234.2-451.9	
Workplace air styrene levels (mg/m^3^)	-			17.8*	12.0-25.0		70.6*	63.0-77	

MA = Mandelic acid; PGA = Phenylglyoxylic acid; creat = creatinine

Data are shown as median [25° percentile-75° percentile] for continuous variables and percentages for categorical variables.

* Ctrl vs low- and high-styrene groups, p value by Mann-Whitney test for continuous variables, and χ^2^ test for categorical variables.

**Figure 2:**
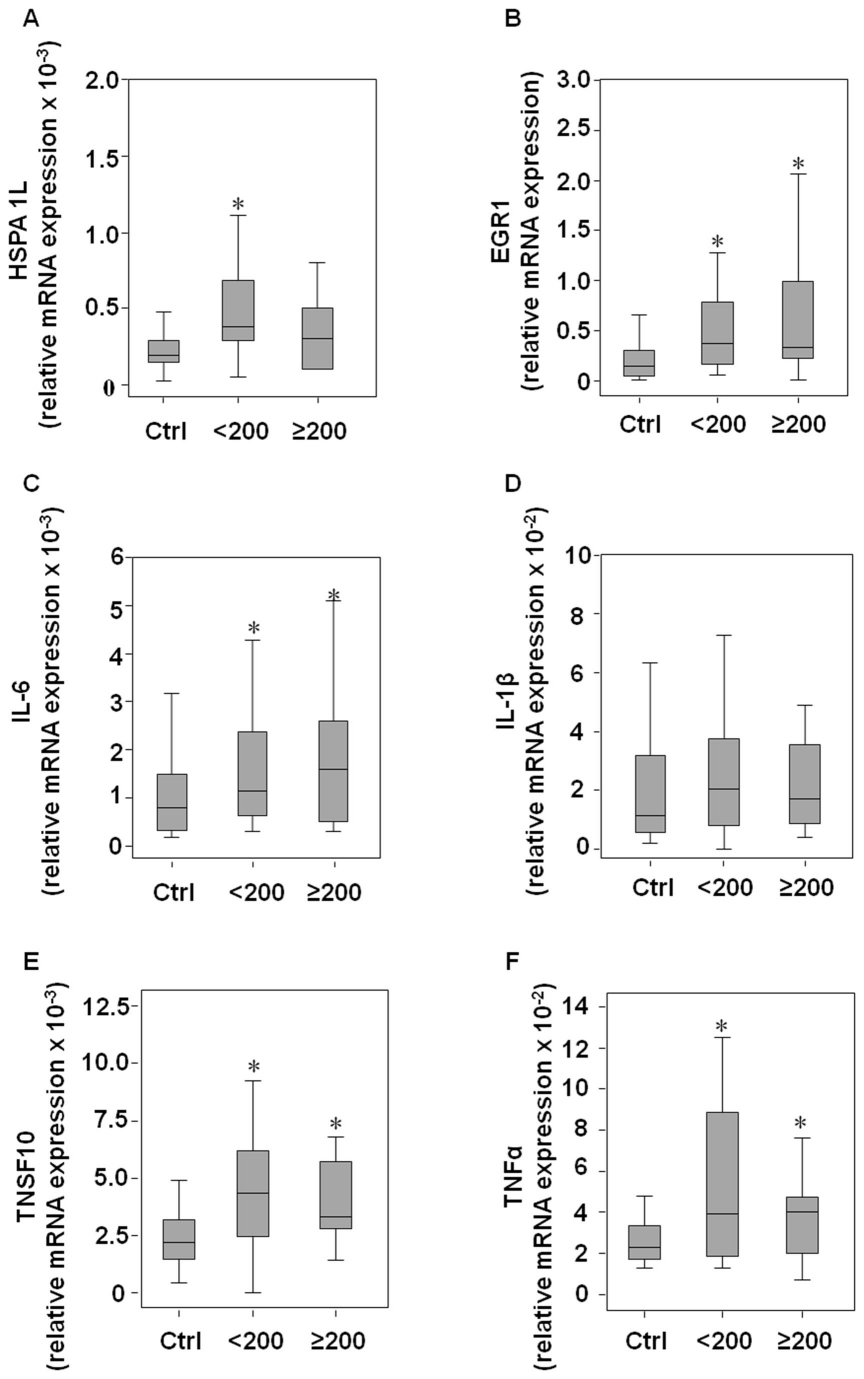
Distribution of stress-related genes in the control, low-styrene and high-styrene groups. Expression of heat shock protein 1L (HSPA1L), early growth response 1 (EGR1), interleukin-6 (IL-6), interleukin-1β (IL-1β), tumour necrosis factor (ligand) superfamily, member 10 (TNSF10) and tumour necrosis factor-α (TNFα) in the low-styrene group (<200 MA+PGA mg/g creat), high-styrene group (≥200 MA+PGA mg/g creat), and control group (Ctrl). Gene-specific product was normalized to GAPDH, expressed as ΔCt, and the relative mRNA expression was calculated using the equation 2^-ΔCt^. Differences among groups were evaluated by Kruskal-Wallis test with post hoc Mann-Whitney test. *Ctrl group vs low- and high-styrene groups, p<0.05.

**Figure 3:**
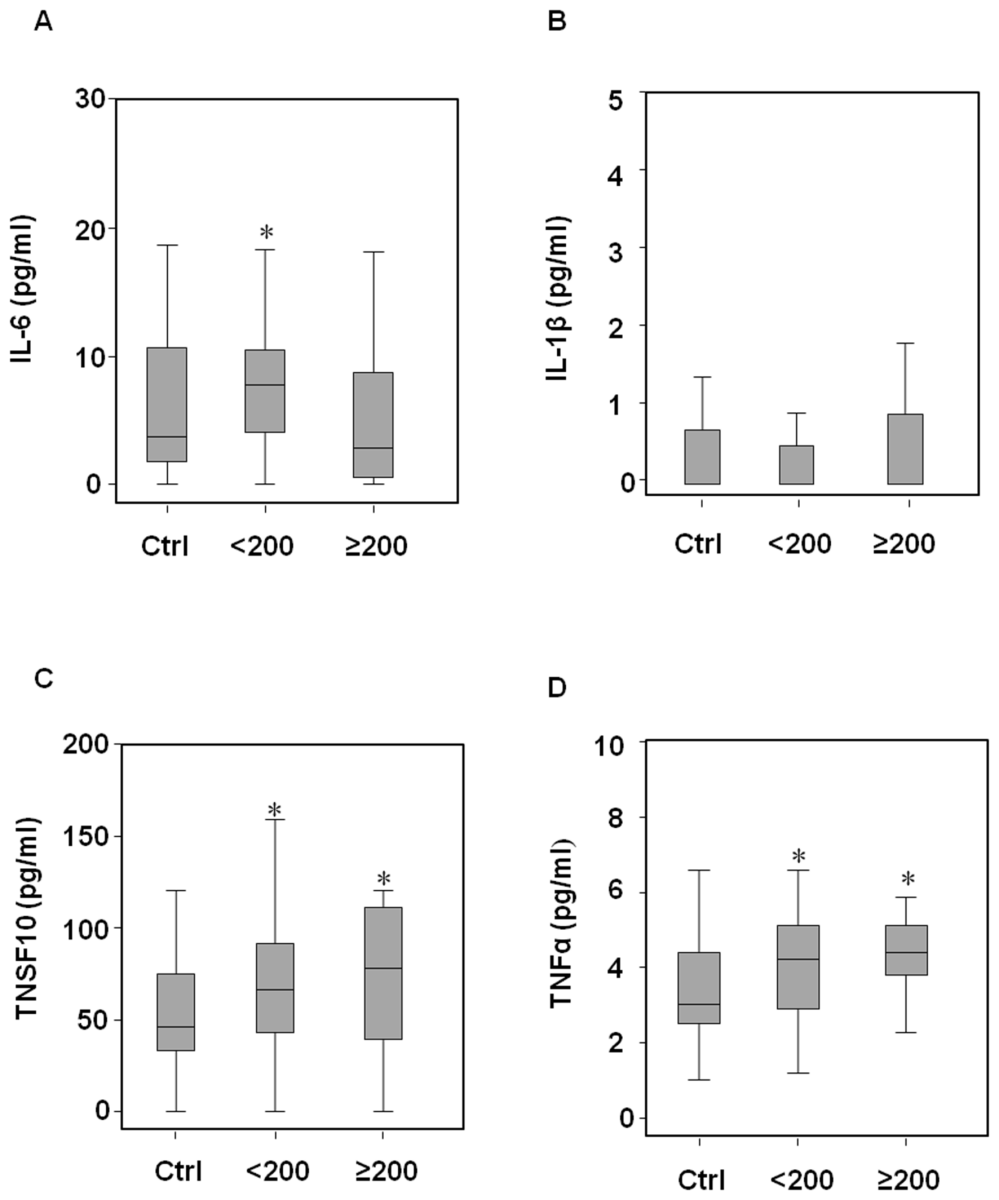
Distribution of cytokines released in serum of control subjects, low-styrene and high-styrene workers. Concentration of interleukin-6 (IL-6), interleukin-1β (IL-1β), tumour necrosis factor (ligand) superfamily, member 10 (TNSF10) and tumour necrosis factor-α (TNFα) in the low-styrene group (<200 MA+PGA mg/g creat), high-styrene group (≥200 MA+PGA mg/g creat), and control group (Ctrl). Cytokines were quantified in serum by ELISA kit and expressed as pg/ml. Differences among groups were evaluated by Kruskal-Wallis test with post hoc Mann-Whitney test. *Ctrl group vs low- and high-styrene groups, p<0.05.

**Figure 4:**
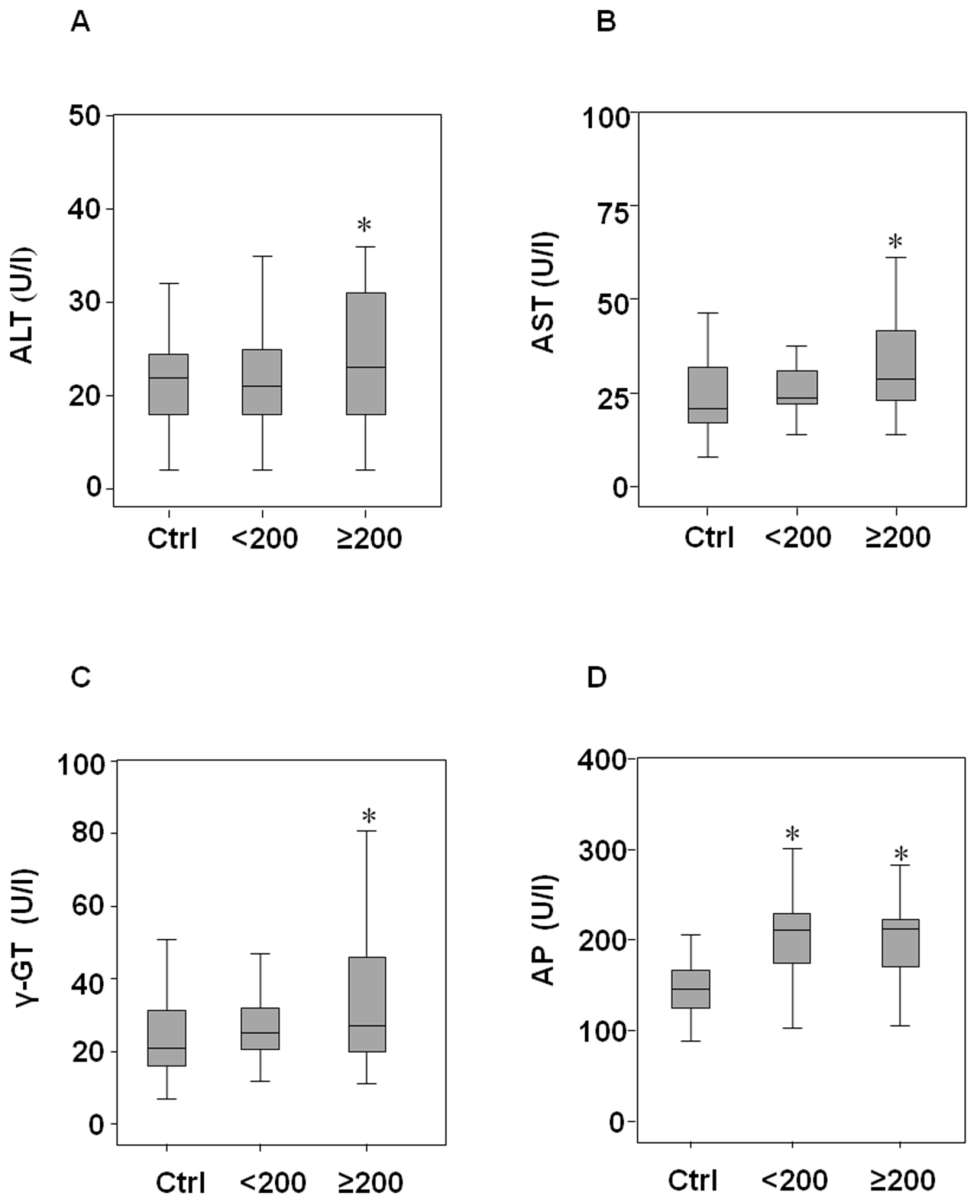
Distribution of hepatic biochemical parameters in control, low-styrene and high-styrene workers. Alanine transaminase (ALT), aspartate aminotransferase (AST), γ-glutamiltransferase (γ-GT), and alkaline phosphatase (AP) were measured in serum of subjects exposed to low-styrene (<200 MA+PGA mg/g creat), high-styrene (≥200 MA+PGA mg/g creat), and in the control group (Ctrl). Differences among groups were evaluated by Kruskal-Wallis test with post hoc Mann-Whitney test. *Ctrl group vs low- and high-styrene groups, p<0.05.

Recently, gene signatures and biological pathways altered by exposure to occupational and environmental carcinogens have been reported [13]. To determine the genes differently expressed in styrene-exposed subjects compared with the non-exposed group, we used a customized PathwayFinder PCR Array with 84 human genes that are known to play a role in toxicology. The gene expression data are available in the ArrayExpress database (www.ebi.ac.uk/arrayexpress) under accession number E-MTAB-1779. Nine styrene-exposed subjects (5 male and 4 female, median [25°-75° percentile], age 41.6 [36.2-47.3], MA+PGA 297.7 [130.0-350.3] mg/g creat) were randomly selected for gene expression profiling. By comparing gene expression from lymphocytes of non-exposed controls (3 male and 2 female, median [25°-75° percentile], age 37.7 [29.3-44.0], MA+PGA 0.3 [0.1-0.5] mg/g creat), a gene profile was obtained (Figure 1). Six genes, heat shock protein (HSPA1L, fold change 12.7 ± 2.5, *p*=0.04), early growth response 1 (EGR1, fold change 4.9 ± 1.7, *p*=0.05), interleukin-6 (IL-6, fold change 9.9 ± 0.1, *p*=0.05), interleukin-1β (IL1β, fold change 21.4 ± 2.1, *p*=0.02), tumour necrosis factor (ligand) superfamily member 10 (TNSF10, fold change 5.0 ± 0.5, *p*=0.01) and tumour necrosis factor-α (TNFα, fold change 2.4 ± 0.8, *p*=0.05) were significantly upregulated in the styrene exposed group. The selected genes were then detected in the whole study population, and based on urinary concentrations of MA and PGA, the styrene-exposed group was divided into two sub-groups: low-styrene exposed group (MA+PGA <200 mg/g creat) and high-styrene exposed group (MA+PGA ≥200 mg/g creat). Demographic characteristics, styrene exposure parameters, and life style habits including smoking and alcohol consumption are shown in Table 1. When the selected genes were tested on whole population, IL-1β lost its significance, while HSPA1L, EGR1, IL-6, TNSF10, and TNFα were found to be over expressed in styrene exposed groups, even at low exposure (Figure 2). The upregulation of genes encoding stress-related cytokines was further confirmed in serum of subjects by immunodetection. IL-6 levels were found to be higher in low-styrene exposed group while TNSF10 and TNFα in both styrene-exposed groups than in the non-exposed control group. No significant changes in IL-1β levels were found (Figure 3). Changes in cytokine levels were associated with an increase of hepatic markers only in the high-styrene exposed subjects (Figure 4). Except for IL-1β, positive correlations were found between each stress-related gene expression and between genes and urinary styrene metabolites (Figure S1). Multiple regression analysis performed to assess the association between gene expression and styrene exposure (MA+PGA) and adjusted for age, gender, ethnicity, body mass index, smoking and drinking habit, confirmed the finding of the univariate analysis. Gender and body mass index mostly affected the hepatic marker levels. However, ALT and AST levels positively correlated with urinary styrene metabolites (Table 2).

**Table 2:** Multivariate regression analysis with hepatic markers as a dependent variable.

Variables	ALT		AST		γ-GT		AP	
	r^2^ = 0.22, p=0.03		r^2^ = 0.20, p=0.05		r^2^ = 0.16, p=0.17		r^2^ = 0.280, p=0.04	
	**β**	***p***	**β**	***p***	**β**	***p***	**β**	***P***
Age	-0.084	0.510	0.058	0.655	-0.022	0.860	0.012	0.092
Gender	**-0.270**	**0.024**	**-0.290**	**0.017**	-0.150	0.220	**-0.400**	**0.001**
Body mass index	**0.230**	**0.036**	0.164	0.149	**0.270**	**0.023**	0.200	0.055
Ethnicity	0.071	0.560	-0.048	0.701	-0.260	0.690	-0.120	0.280
Smoking	0.150	0.190	0.084	0.493	0.006	0.960	-0.120	0.270
Drinking	0.093	0.470	-0.024	0.849	0.190	0.150	0.120	0.320
Exposure duration	-0.110	0.356	-0.148	0.243	-0.180	0.160	-0.012	0.900
MA+PGA	**0.240**	**0.044**	**0.245**	**0.045**	0.140	0.250	-0.023	0.800

MA = Mandelic acid; PGA = Phenylglyoxylic acid; ALT = alanine transaminase; AST = aspartate aminotransferase; γ-GT = γ-glutamiltransferase; AP = alkaline phosphatase.

Multivariate regression analysis adjusted for age, gender, ethnicity, body mass index, smoking and drinking habit. Significances are highlighted in bold.

## Discussion

Although occupational exposure to styrene has steadily declined over the years due to improved industrial hygiene and more stringent regulations, the number of workers exposed to styrene is still high. In the present study, we evaluated the effect of occupational styrene exposure on the expression of genes involved in toxicology. Several studies have shown that styrene itself, or its metabolites, can induce direct hepatotoxicity by covalent binding to intracellular molecules or by oxidative stress [9,14,15,16]. Excessive production of reactive oxygen species (ROS) affects liver cellular function. This may result in an alteration of gene expression/profile and promote or protect cell death [17,18]. Here, we found that, even at low concentration, styrene induced expression of the inflammatory mediators HSPA1L, EGR1, IL-6, TNSF10 and TNFα, which was reported to be involved in tissue injury and regeneration [19]. Styrene-dependent upregulation of the heat shock response observed in styrene-exposed subjects is consistent with earlier findings that demonstrated the ability of toxicants to induce the heat shock response involved in cytoprotective and cytotoxic activities [20,21]. HSPA1L protein is accepted as a cytoprotective agent [22,23] involved in cellular maintenance and repair mechanisms, including its role as an anti-inflammatory protein [24].

Inflammatory gene expression plays a pathological role in acute and chronic hepatic inflammation promoting liver repair by inducing protective mechanisms to limit collateral tissue damage. The TNFα and IL-6 were shown to be important mediators of the regenerative process of hepatocytes [25,26,27,28]. The regenerative response including TNFα was reported to be regulated by the EGR1 [29,30]. EGR1 is a transcription factor rapidly induced by many growth and differentiation signals that regulate inflammatory gene expression [19,31]. EGR1 was found to be expressed in liver in response to acute carbon tetrachloride (CCl_4_) exposure which contributed to the regulation of a large number of genes involved in the regenerative response [19,29]. In our system, EGR1 gene expression induced by styrene exposure was found to be positively correlated with the expression of inflammatory cytokines.

Pro-inflammatory cytokines such as IL-6 and TNFα are released into bloodstream both from the liver and from distal sites during hepatic toxic injury [32], and can both induce apoptosis and amplify cell growth. In the contest of liver injury, it is important to consider the ability of these mediators to exacerbate cell damage by amplifying the inflammatory process. This can occur by the initiation of an aggressive inflammatory process through the recruitment and activation of neutrophils and monocytes at the site of damage [33,34]. Indeed, TNFα and IL-1β signalling pathway can induce NF-kB transcription, which in turn drives transcription of IL-6, IL-8 and TNFα itself [17,35]. The increased expression of cytokines observed at low styrene exposure was associated with a slight increase of transaminases in highly exposed workers, even though the hepatic markers were within the normal range. The effect of styrene exposure on hepatic marker levels is controversial. No effect of styrene exposure on the level of transaminases was previously reported [36], while, evidence of hepatocellular toxicity induced by styrene has been shown at high exposure (300-400 mg/kg) [37]. Several epidemiological studies reported increased serum levels of hepatic transaminases in workers exposed to relatively high concentration of styrene [38,39,40], while lower exposure to styrene was found to be associated with mild subclinical injury, characterized by diminished clearance of conjugated bilirubin [9].

In conclusion, our study reports that chronic exposure to styrene at levels below TLV-TWA_8_ induced EGR1 expression as event in response to styrene exposure, preceding IL-6 and TNFα expression, which are involved in the regulation of several genes and/or pathways involved in liver regeneration after hepatotoxic exposure. Pointing out that occupational exposure to styrene could still result in effects that may be involved in the development of diseases.

## Supporting Information

Figure S1 **Correlations among stress-related gene expression and between gene expression and urinary styrene metabolites.** Spearman’s correlation coefficient analysis. *p<0.05, **p<0.01.
(TIF)
