# Supplementary figures and images for: Occupational Styrene Exposure Induces Stress-Responsive Genes Involved in Cytoprotective and Cytotoxic Activities
Source: PLoS One. 2013 Sep 23;8(9):e75401. doi: 10.1371/journal.pone.0075401 (PMC3781025; doi:10.1371/journal.pone.0075401)

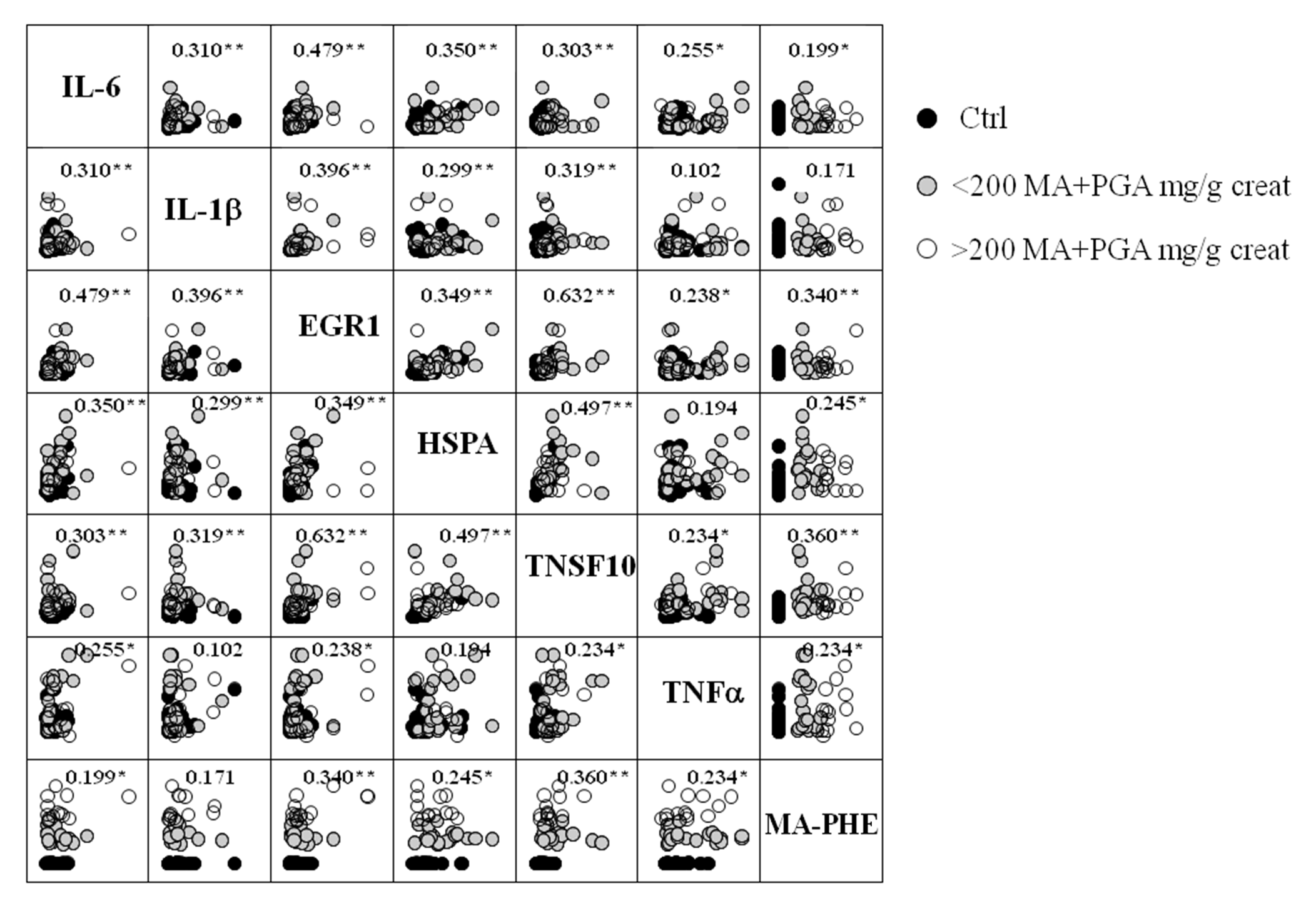

Supplement: Figure S1 — Correlations among stress-related gene expression and between gene expression and urinary styrene metabolites. Spearman’s correlation coefficient analysis. *p<0.05, **p<0.01. (TIF) [file pone.0075401.s001.tif]
